# Draft mitochondrial genomes of *Hirudo medicinalis* and *Hirudo verbana* (Annelida, Hirudinea).

**DOI:** 10.1080/23802359.2016.1157774

**Published:** 2016-03-28

**Authors:** Anastasia Nikitina, Vladislav Babenko, Tatyana Akopian, Dmitriy Shirokov, Valentin Manuvera, Alexey Kurdyumov, Elena Kostryukova, Vassili Lazarev

**Affiliations:** a Department of Molecular Biology and Genetics, Federal Research and Clinical Center of Physical-Chemical Medicine of Federal Medical Biological Agency, Moscow, Russian Federation;; b Department of Biological and Medical Physics, Moscow Institute of Physics and Technology, Dolgoprudnyi, Russian Federation

**Keywords:** *Hirudo medicinalis*, *Hirudo verbana*, mitochondrial genome

## Abstract

Here we present two incomplete mitochondrial genome sequences of *Hirudo medicinalis* and *Hirudo verbana* (Annelida, Hirudinea). The corresponding sequences are 14,729 and 14,604 base pairs in length. They contain all mitochondrial genes (13 protein-coding genes, 22 tRNAs and two rRNAs) but lack the non-coding region. Nevertheless, the robust reconstruction of their phylogenetic relationships presented here reveals distinct separation of both leeches from other annelids and at the same time relatively high dissimilarity between each other.

*Hirudo medicinalis,* one of the species more commonly known as medicinal leech, has been used in various medical practices for centuries. Nowadays being approved by US FDA as prescription medical device in 2004, it has several clinical applications such as reconstructive surgery (Porshinsky et al. 2011), at the same time, attracting research as widely used model organism in fields like neurobiology (Kristan et al. 2005) . In 2007 Sidall et al. (2007) demonstrated that commercially available leeches marked as *H. medicinalis* were actually *H. verbana,* so closely related species that there are concerns as to whether they are in fact different species (Hildebrandt & Lemke 2011). Since morphology, geographical distribution and incomplete reproductive isolation (Petrauskienė et al. 2009) are too controversial to elucidate the phylogenetic relationships of these leeches, mitochondrial DNA (mtDNA) sequences might be invaluable to this task. In this study, we report two draft mitochondrial genomes of *H. medicinalis* and *H. verbana* lacking only one non-coding region each.

The specimens were provided by HIRUD I.N. Ltd. (Balakovo, Saratov Region, Russia). The collection of *H. medicinalis* took place at the pond near Volkovo, Saratov region, Russia (51°91′03″, 47°34′90″) and *H. verbana –* at the lake Manych, Stavropol Krai, Russia (46°01′09″, 43°48′21″). Total genomic DNA was isolated from muscle tissue, and amplicons corresponding to mtDNA were generated by PCR and sequenced using Ion Proton (Life Technologies, Carlsbad, CA). Primary assembly was conducted by Newbler 2.6 (Life Technologies, Carlsbad, CA) and gaps were filled via Sanger sequencing using ABI Prism Genetic Analyzer 3730XL (Applied Biosystems, Waltham, MA). The annotation was performed by web-based tool MITOS (Bernt et al. 2013) and then manually corrected by comparison with complete mitochondrial genomes of other annelids.

The sequences of mtDNA of *H. medicinalis (*14,729 bp) and *H. verbana* (14,604 bp) reported here were deposited in GenBank under the accession numbers KU672396 and KU672397, respectively. Each of them comprised 13 protein-coding genes, 22 tRNAs and two rRNAs all encoded on the same strand. The gene order shown in Table 1 was consistent with other members of Hirudinea subclass. However, the non-coding regions of both leeches located between *tRNA-Arg* and *tRNA-His* appeared to be longer and more complex compared with those of close relatives, hence were only partially assembled and included in the sequences resulting in two almost complete mitogenomes. The annotation of the latter is represented in Table 1 along with predicted start and stop codon usage which was found to be identical between *H. medicinalis* and *H. verbana.* They employed three different start codons: ATG (*ND1, ND2, ND3, ND4, ND6, COX1, CYTB, ATP6* and *ATP8*), GTG (*ND5, ND4L* and *COX2*) and TTG (*COX3*). Stop codon of choice for these mitochondria was TAA, although six genes (*ND2, ND4, ND6, COX1, COX3* and *ATP8*) utilized termination codon T–, completed via polyadenylation.

**Table 1.** The annotation of mtDNA sequences of *H. medicinalis* and *H. verbana.*

	*Hirudo medicinalis*		*Hirudo verbana*				
Gene	Location, bp	Size, bp	Location, bp	Size, bp	Anticodon	Start codon	Stop codon
*tRNA-His*	93–153	61	183–243	61	GTG		
*ND5*	154–1857	1704	244–1953	1710		GTG	TAA
*tRNA-Phe*	1857–1918	62	1953–2013	61	GAA		
*tRNA-Glu*	1919–1972	54	2022–2068	47	TTC		
*tRNA-Pro*	1977–2037	61	2072–2132	61	TGG		
*tRNA-Thr*	2039–2097	59	2134–2192	59	TGT		
*ND4L*	2098–2385	288	2193–2480	288		GTG	TAA
*ND4*	2379–3711	1333	2474–3806	1333		ATG	T–
*tRNA-Cys*	3721–3780	60	3816–3875	60	GCA		
*tRNA-Met*	3781–3843	63	3876–3939	64	CAT		
*s-rRNA*	3844–4583	740	3940–4677	738			
*tRNA-Val*	4584–4642	59	4678–4736	59	TAC		
*l-rRNA*	4643–5782	1140	4737–5880	1144			
*tRNA-Leu*	5783–5842	60	5881–5940	60	TAG		
*tRNA-Ser*	5843–5909	67	5941–6007	67	TGA		
*tRNA-Ala*	5910–5971	62	6008–6069	62	TGC		
*tRNA-Leu*	5972–6032	61	6070–6130	61	TAA		
*ND1*	6033–6953	921	6131–7051	921		ATG	TAA
*tRNA-Ile*	6952–7013	62	7050–7111	62	GAT		
*tRNA-Lys*	7014–7075	62	7112–7173	62	TTT		
*ND3*	7076–7420	345	7174–7518	345		ATG	TAA
*tRNA-Ser*	7419–7475	57	7517–7573	57	TCT		
*ND2*	7476–8460	985	7574–8561	988		ATG	T–
*COX1*	8461–9994	1534	8562–10,095	1534		ATG	T–
*tRNA-Asn*	9995–10,057	63	10,096–10,159	64	GTT		
*COX2*	10,058–10,741	684	10,160–10,843	684		GTG	TAA
*tRNA-Asp*	10,740–10,802	63	10,842–10,904	63	GTC		
*ATP8*	10,803–10,953	151	10,905–11,055	151		ATG	T–
*tRNA-Gly*	10,954–11,011	58	11,056–11,114	59	TCC		
*tRNA-Tyr*	11,012–11,071	60	11,114–11,172	59	GTA		
*COX3*	11,089–11,869	781	11,189–11,969	781		TTG	T–
*tRNA-Gln*	11,870–11,938	69	11,970–12,038	69	TTG		
*ND6*	11,939–12,398	460	12,039–12,498	460		ATG	T–
*CYTB*	12,399–13,541	1143	12,499–13,641	1143		ATG	TAA
*tRNA-Trp*	13,543–13,604	62	13,643–13,704	62	TCA		
*ATP6*	13,663–14,388	726	13,763–14,467	705		ATG	TAA
*tRNA-Arg*	14,380–14,427	48	14,477–14,524	48	TCG		

In order to infer the phylogenetic relationships of these leeches, seven complete mitochondrial DNA sequences of other annelids were downloaded from NCBI GenBank, six of which represented Hirudinea subclass (*Hirudo nipponia, Whitmania laevis, Whitmania pigra, Whitmania acranulata, Erpobdella octoculata* and *Poecilobdella manillensis*) and one (*Metaphire californica*) belonged to Oligochaeta subclass and was used as the outgroup. Circular mitogenomes were linearized to start with *tRNA-His* and end with *tRNA-Arg* for alignment and following phylogenetic analysis. The resulting tree drawn to scale is shown in Figure 1. Interestingly, *H. medicinalis* and *H. verbana* form a separate clade from all other species of Hirudinea subclass. It is also worth pointing out that corresponding branch lengths within this clade suggest enough divergence for these two leeches to be classified as different species.

**Figure 1.**
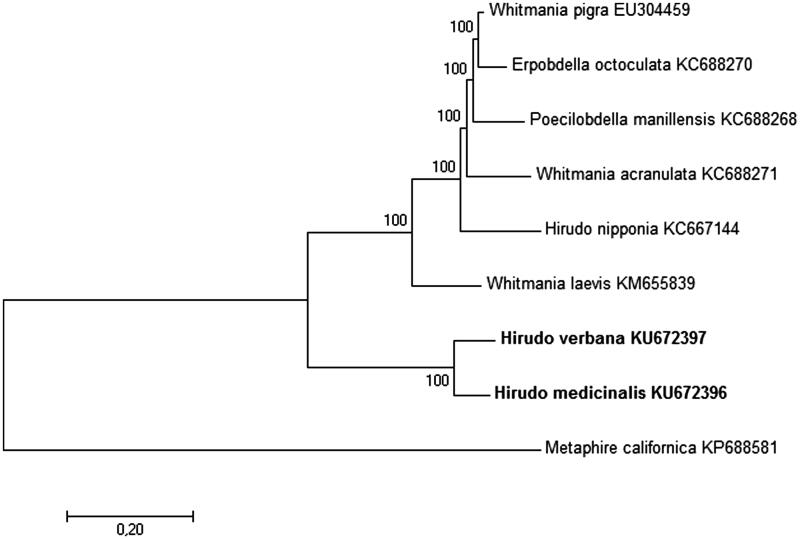
The phylogenetic relationships of *H. medicinalis* and *H. verbana* (both shown in bold). The analysis was performed using Maximum Likelihood method based on the Tamura 3-parameter model (Tamura 1992) in MEGA7 (Kumar et al. 2012). Metaphire californica was used as an outgroup. The robustness of each node is represented by a bootstrap value obtained by 500 steps. The scale bar corresponds to a number of substitutions per site. Each NCBI accession number of the sequence used to construct the tree is shown next to corresponding taxa name.
